# Fungal Effectoromics: A World in Constant Evolution

**DOI:** 10.3390/ijms232113433

**Published:** 2022-11-03

**Authors:** Jewel Nicole Anna Todd, Karla Gisel Carreón-Anguiano, Ignacio Islas-Flores, Blondy Canto-Canché

**Affiliations:** 1 Unidad de Biotecnología, Centro de Investigación Científica de Yucatán, A.C., Calle 43 No. 130 x 32 y 34, Colonia Chuburná de Hidalgo, Mérida C.P. 97205, Yucatán, Mexico; 2 Unidad de Bioquímica y Biología Molecular de Plantas, Centro de Investigación Científica de Yucatán, A.C., Calle 43 No. 130 x 32 y 34, Colonia Chuburná de Hidalgo, Mérida C.P. 97205, Yucatán, Mexico

**Keywords:** fungal effectors, effector interactions, effector biology, effectoromics, plant disease

## Abstract

Effectors are small, secreted molecules that mediate the establishment of interactions in nature. While some concepts of effector biology have stood the test of time, this area of study is ever-evolving as new effectors and associated characteristics are being revealed. In the present review, the different characteristics that underly effector classifications are discussed, contrasting past and present knowledge regarding these molecules to foster a more comprehensive understanding of effectors for the reader. Research gaps in effector identification and perspectives for effector application in plant disease management are also presented, with a focus on fungal effectors in the plant-microbe interaction and interactions beyond the plant host. In summary, the review provides an amenable yet thorough introduction to fungal effector biology, presenting noteworthy examples of effectors and effector studies that have shaped our present understanding of the field.

## 1. Introduction

Effectors are molecules that provide an evolutive advantage to organisms as they complete their life cycles and thrive in their respective niches. Effectors are traditionally defined as molecules that manipulate host cell structure and function, thereby facilitating infection (virulence factors or toxins) and/or triggering defense responses (avirulence factors or elicitors) [1]. We define effectors as molecules of diverse functionality and molecular nature that influence organisms’ interactions with each other, usually to the benefit of the organism using them. The effector gene products are produced by one organism but they mainly function in another, resulting in the alteration of host cell structure and function [1,2,3]. Effectors were first discovered in the pathogen-plant interaction between a biotrophic fungus and its plant host but have since been widely described in different pathosystems involving biotrophic, hemibiotrophic, and necrotrophic fungi [4,5,6]. Biotrophic organisms require live hosts to complete their life cycle, and their effectors allow them to stealthily enter and remain in the host while avoiding recognition and suppressing the host’s defenses [7,8,9]. Necrotrophs, which are heavily dependent on cell wall-degrading enzymes and phytotoxins to infect hosts also use effector molecules [10,11,12].

In each ecological interaction, communication exists between effectors and receptors that shape the outcome of the interaction. Although widely associated with pathogenicity, effectors have been discovered in plant-beneficial organisms such as mutualist mycorrhizal species and endophytes [13,14,15,16] and the study of their roles in microbe-microbe interactions is gaining traction in the field of effector biology [17,18]. These molecules are ubiquitous in microorganisms, and the effectorome complement of each organism is highly specialized and greatly affects its lifestyle, as both gene gain and loss of certain effectors have implications for pathogen virulence and host adaptation [19,20,21,22]. Effectors have even shed their “microbial” association and have been identified in larger organisms [23,24,25,26] and are also defense molecules used by plants in their interactions with invaders [27,28]. It is now more evident than ever before that effectors participate in diverse associations between organisms that transcend the boundaries of taxonomic kingdoms and lifestyles, making them cornerstones of biological interactions.

Effectors can be classified in many ways: according to their molecular nature, effector molecules can be proteins [29,30], secondary metabolites [31,32,33], or small RNAs [34,35]. Apart from their molecular nature, effectors can be classified based on their site of action in the host plant i.e., apoplastic (extracellular) or intracellular; the latter comprising effectors targeting host proteins in the cytoplasm and cell organelles [1,36,37,38]. Some effectors may also be race-specific i.e., only found in some strains or isolates of a species [39,40] while other effectors are common to the genomes of different closely or distantly related species and are termed “core effectors” [41,42]. Another type of classification for effectors is based on their interactions with organisms or host specificity [43] since effectors participate in more than one type of interaction, e.g., in both plant-microbe and microbe-microbe interactions, while other effectors are interaction-specific.

**Figure 1:**
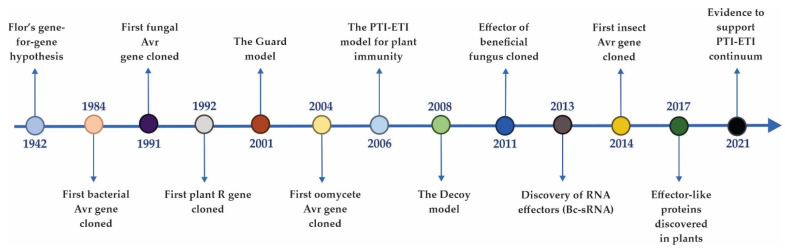
A timeline of some noteworthy milestones that shaped Effector Biology.

Since the hypothesis of pathogen Avr genes by Flor in 1942, our knowledge of these molecules has greatly expanded, with various models produced to explain effector interactions with their receptors and the application of molecular biology techniques to elucidate the functions of chosen microbial effectors (Figure 1), but the study of effectors is far from black and white. The present review gives a comprehensive summary of the pioneering literature as well as, disruptive findings regarding effector molecular nature, localization, taxonomic distribution, and the types of interactions involving effectors. The associated changes that have occurred within effector identification methods over the years and novel bioinformatic tools are also briefly discussed. Remarkable effector reviews that summarized significant findings regarding effector identification, evolution, effector functions, host targets, and Avr-R interactions were published in 2009 [3,30,44] followed by noteworthy publications in the last decade that, in large part, have explored newly discovered effector functions and how they undermine plant defense [4,5,36,37,45,46]. To our knowledge, this review is the first of its kind to present recent updates in effector biology and demonstrate how they may differ from previously established dogmas in the field. Furthermore, a wider range of effector topics is addressed than in previous publications, and interesting findings are organized according to each theme. Finally, a comprehensive table compares the “past versus present” ideas surrounding these themes. In summary, the objective of the review was to demonstrate how effector concepts have evolved over the last three decades, challenging the initial definition of an effector.

## 2. Fundamentals of Effector Biology

Effectors were first called ‘avirulence factors’ as described by the botanist Flor in the 1940s [47]; genes called Avr were used by pathogens for which a cognate ‘R’ or resistance gene existed in plants resistant to the particular pathogen. This was dubbed the ‘gene-for-gene hypothesis and in Flor’s work, it was applied to the fungus *Melampsora lini* and the plant host, the flax plant, *Linum usitatissimum*. Though the original discovery was made between these two organisms it was found to apply to other pathogens in interaction with their plant hosts, although it is unlikely that the gene-for-gene interaction is the most common interaction found between effectors and targets [48]. Resistant plants evolved with resistance gene products to recognize pathogen Avrs and curtail the infection; this phenomenon is discussed later in this section. Conversely, gene products belonging to the host which promote the infection are encoded by susceptibility (S) genes, where negative regulators of plant immunity can be found [49]. Triggering susceptibility is fundamental for the compatible pathogen-host interaction, ultimately resulting in the development of disease. Most effectors characterized to date, target positive regulators of host immunity, interfering with their functionality in host defense [46].

Regarding plant immunity, MAMPs and effectors are the major elicitors of host defense; conserved molecules called MAMPs are the elicitors of host defense mechanisms (deposition of callose, induction of pathogenicity-related proteins, oxidative burst) that are collectively referred to as MAMP-triggered immunity (MTI). MTI is induced upon the recognition of MAMPs by plasma membrane-localized pattern recognition receptors (PRRs). These mechanisms are also induced and potentiated during another defense tier called Effector-triggered immunity (ETI) which is based on a unique protein-protein interaction: the recognition of the ‘Avr’ protein of the pathogen by the ‘R’ protein of the host. Many R proteins are intracellular and belong to the family of nucleotide-binding and leucine-rich repeat domain (NLR) proteins [50,51]. Regarding the pathogen, microbes most likely evolved with effectors to overcome the basal immunity response of the plant; when PTI and ETI are compromised in the host due to effector deployment, this phenomenon is referred to as “Effector-triggered susceptibility” [50,52,53]. The defining characteristic of ETI is a form of programmed cell death induced in the plant called the hypersensitive response (HR) and the induction of systemic acquired resistance (SAR), which prevents further propagation of the pathogen. ETI induced by the Avr-R protein interaction is essential to the incompatible pathogen-host interaction, where the pathogen fails to cause disease. Pathogen Avr genes have virulence functions that aid in disease development, e.g., the *Cladosporium fulvum* Avr effectors Avr2 [54] and Avr4 [55] which suppress MTI. Avr-Pik is another example of a fungal Avr whose virulence function is known; Avr-Pik binds and stabilizes the host protein OsHIPP20, a product of a susceptibility gene that promotes *Magnaporthe oryzae* infection [56]. Some Avrs suppress ETI mediated by other Avrs, like the AvrLm4-7 effector of *Leptoshaeria maculans* that suppresses the AvrLm3-Rlm3 mediated resistance response in *Brassica napus* [57]. The virulence functions of most Avrs remain unknown.

The Avr and R genes of pathogens and plants are under high selective pressure to evolve; mutations and/or gene losses and gains of the Avr allow pathogens to evade recognition while the plant (at a much slower rate) evolves new R genes to maintain the ability to recognize the Avrs in order to protect itself [50]. The direct interaction between Avr-R is one of a few types of effector-target interactions in the plant. The guard hypothesis suggests that R genes exist that guard many possible targets [58], while in the decoy model the Avr protein has an indirect target, a decoy, and the R protein is alerted by this interaction [59]. The integrated decoy model explains how some R proteins (NLR receptors) have evolved to integrate additional decoy domains that act as a sensor or bait for Avrs; in this interaction, the bait binds to the effector, and another receptor interacting with the bait triggers defense signaling [60,61]. In another effector interaction theory, not every molecule that an effector will interact with is its true target; effector ‘helpers’ exist which aid in signal transduction or signal amplification like “helper” NLRs which interact with true “sensor” NLRs [62,63]. Other helpers may be cofactors, chaperones that aid in protein folding, or transporters, among other functions, that ultimately aid in host susceptibility or defense [64,65].

The strict separation of the MTI-ETI dichotomy and the zig-zag model has been challenged for various reasons: (1) the zig-zag model was established for biotrophic pathogens, (2) some effectors are broadly conserved and can therefore be considered MAMPs, and (3) the stages of MTI and ETI are not static [66]. A more inclusive model called the “invasion model” was therefore proposed to rename the players involved. All immunogenic molecules (effectors and elicitors/MAMPs) are described as invasion patterns (IPs) perceived by plant IP receptors (IPRs), leading to an IP-triggered response (IPTR) [66]. The more recent “iceberg model” suggests that both resistance (R) and susceptibility (S) targets are monitored by NLRs that act in “interaction units”; the majority of interaction units are silent (due to effectors that suppress host immunity) and make up the larger portion of the iceberg which is under the surface and unseen, while the visible tip of the iceberg consists of those interaction units (effector and receptor interactions) that are able to induce host defense resulting in the resistant phenotype [53].

The latest developments in effector biology revolve around the MTI-ETI dichotomy as it is now clearer than ever that the two are more interrelated than they are distinct. MTI and ETI share many of the same components; recently it was observed that TIR signaling mutants (domains found in NLR resistance proteins involved in ETI) displayed compromised MTI and overexpression of TIR genes amplified the MTI defense response [67]. In the same vein, another study found that ETI’s hypersensitive response is enhanced by MTI PRRs and that effective host resistance is mediated by MTI and ETI working in concert [68].

## 3. Effector Identification: Past and Present

The first 80 years of effectoromics were challenge-ridden due to the lack of homology present among effector molecules and also due to discrepancies in effector identification methods. Certain criteria have been established to identify effector candidates; researchers either prepare in-house tailored pipelines for effector identification or use already established programs that are based on common effector characteristics. Effector candidature is usually determined based on sequence length ≤ 300 amino acids; cysteine richness (>2% cysteine content or >4 cys residues), presence of a secretory signal peptide; absence of transmembrane domains; higher expression in interaction with the host; limited taxonomic distribution with no or limited sequence similarity to other organisms and encoded by genes with long intergenic regions or in gene-sparse, repeat-rich chromosomes [30,36,69,70,71].

The number of validated effectors in recent times has increased from 96 in 2018 to more than 300 in 2022 [69,72,73,74]. This has occurred due to advances in ‘omics’ studies and the development of bioinformatic tools. Bioinformatic tools used for the prediction of effector characteristics include SignalP (https://services.healthtech.dtu.dk/service.php?SignalP-5.0; [75]), for the detection of signal peptides; TargetP (https://services.healthtech.dtu.dk/service.php?TargetP-2.0; [76]), LOCALIZER (http://localizer.csiro.au/; [77]), and WoLF PSORT (https://wolfpsort.hgc.jp/; [78]) for the determination of effector localization and DeepTMHMM (https://dtu.biolib.com/DeepTMHMM; [79]), for the prediction of transmembrane domains where the absence of said domains is preferred. Databases of experimentally validated proteins with roles in pathogen virulence have been especially useful for effector prediction. The database PHI-base which has a compendium of genes involved in plant-pathogen interactions allows users to compare their effector candidates with those homologs in the database using PHI-BLAST (http://phi-blast.phi-base.org/; [80]). These bioinformatic prediction tools and databases have supported effector predictions on high, medium, and low-throughput screenings for effectors. In the last decade, a turning-point for effector prediction came with the creation of software based on machine-learning (ML) methods that predict effectors based on sequence characteristics shared among experimentally validated effectors. Notably, Sperschneider and colleagues created the EffectorP series, the most commonly used machine learning algorithm for fungal effector prediction [74,81,82].

EffectorP 1.0 is a Naïve Bayes machine learning predictor that was trained with 58 true fungal effectors from 16 fungal species. The negative dataset (14,143 proteins) was constructed based on the secretomes (total set of secreted proteins) of 16 fungal species, filtering the known effectors and homologs. Although EffectorP 1.0 improved fungal effector prediction from secretomes, it was trained with a negative dataset including both undiscovered effectors and non-effectors [81]. EffectorP 2.0 was then trained with 94 secreted true effectors from 23 fungal species and the negative dataset (21,840 proteins) was constructed initially as it was for EffectorP 1.0, but with 23 fungal pathogen secretomes, after which EffectorP 1.0 was applied to exclude potentially undiscovered effectors. To this output, the secretomes from 27 non-pathogenic saprophytes, and 10 animal-pathogenic fungal secretomes were added. As a result, the updated EffectorP 2.0 was successful in decreasing by 40% the number of effector candidates from the negative set that was from fungal plant symbionts and saprophytes vs. EffectorP 1.0 [82]. EffectorP 2.0 has been the most frequently used fungal effector predictor in recent years. However, reports about effectors in non-pathogenic fungi are ever increasing [83,84], and these effectors may be excluded during effector mining, even by EffectorP 2.0. The latest EffectorP algorithm, version 3.0, classifies effectors according to apoplastic and cytoplasmic localization [74].

Another recent predictor of fungal effectors, Effhunter, is a pipeline written in Perl/Bioperl language that retrieves effector candidates that strictly meet the canonical or conventional effector criteria: secreted (signal peptide presence), small size (<400 amino acids), cysteine-rich (>4 residues) and no transmembrane domain [69]. Effhunter is a suitable effector predictor, possessing the highest F1 score for identifiers of canonical fungal effectors. Regarding oomycetes, the ML algorithm EffectorO identifies proteins unique to one species or genus of oomycetes and was able to predict larger effectoromes than previously estimated for *Bremia lactucae* and *Phytophthora infestans* [73].

Many effectors evade the current predictors because of their non-conventional nature; these effectors called non-canonical effectors (NCEs) constitute approximately 90% of an organism’s effectorome according to WideEffHunter (Carreón-Anguiano et al. submitted). Since some effectors do not meet all the criteria used to define them, researchers in effectoromics face a great challenge during in silico effector identification. To help researchers prioritize the most important criteria for selecting or ranking effectors, authors of WideEffHunter *in silico* characterized 314 true (validated) fungal and oomycete effectors. The ranking of the criteria was as follows: the absence of glycosylphosphatidylinositol anchors (GPI), 96.5%; the absence of transmembrane domains (TMD), 91.1%; sequence length < 400 amino acids, 89.4%; the presence of a signal peptide, 85%; extracellular localization, 71.6%; >4% Cys content, 54.4%. The determination of the importance or weight of each of these effector criteria will be beneficial for future effector predictions and the selection of candidates for further characterization in the lab.

NCE characteristics have been shown to vary across proteins; some are larger than 400 amino acids (Ace1: [85]), lack a signal peptide (VdIsc1: [86]), have low cysteine contents (AvrLm1: [87]) while others show high similarity to other effector sequences or are conserved among species e.g., ceratoplatanins [88], the latter being easier to recognize by pipelines that integrate homology as one of the effector identification characteristics. These non-conventional effectors require creative strategies for their identification, like the use of effector-related motifs. The motifs RXLR, LFLAK, Y/F/WxC, and CRN motifs are commonly used for identifying effectors in oomycetes, but recently, Zhao et al., (2020) used this strategy on the fungus *Puccinia graminis* and found 719 RXLR, 19 CRN, and 138 Y/F/WxC new effector candidates [89]. Recently, the Predector [90] pipeline, which was created for ranking effector candidates, was able to identify MoCDIP8, a non-canonical *Magnaporthe oryzae* effector with two predicted transmembrane domains and no signal peptide; the predictors EffHunter, EffectorP 1.0 or EffectorP 2.0 fail to recognize this protein as an effector. Predector also recognizes MoCDIP13 which is retrieved by EffectorP 1.0 but not by Effhunter and EffectorP 2.0.

Admittedly, it would be difficult for any singular algorithm to correctly identify all the effectors of an organism’s effectorome, but this is an exciting era where improvements in the current algorithms and newer algorithms are increasing the number of true effectors identified per organism. Hundreds of effector candidates are being identified from omics projects, necessitating strategies for prioritizing candidates for functional analysis.

## 4. Effector Conservation: Effectors Shedding the “Species-Specific” Label

The genes that encode effectors are commonly referred to as “compartmentalized” in gene-sparse, repeat-rich regions of the accessory chromosomes of the genome which facilitate their “high-speed” evolution. These regions are characterized by high mutation rates and chromosomal rearrangements [71,91,92,93]. A “two-speed” genome model proposes that genome organization could be broadly divided into two parts: the core genome which holds essential genes that are protected from high rates of mutation while the accessory genome has the effectors which require rapid evolution [92,94,95]. This two-speed genome arrangement has been documented, for example, in the fungi *Verticillium dahliae* [96], *Leptosphaeria maculans* [97], and *Colletotrichum higginsianum* [98]. The accessory or “dispensable” chromosomes are not found in all races or strains of a pathogen; they are referred to as strain- or pathotype-specific chromosomes [99]. However, not all genomes exhibit compartmentalization of virulence genes, and gene distribution is rather homogenous in “one-speed” genomes [100,101].

Effectors were once commonly referred to as race-specific or lineage-specific, taking part in interactions that are unique to one host or a limited host range [40,102,103]. Some effectors are more conserved than others and can be found among all the strains of a pathogen and are referred to as “core” effectors [41,104]. The term has been extended to those effectors that are also found in related species of a taxonomic family [42]. It is hypothesized that effector genes that are conserved among pathogens and reside in core genome regions are most likely to hold indispensable virulence functions. As such, they are not under the same rate of diversifying selection as their race-specific effector counterparts [105]. Various core effectors have been identified from the biotrophic fungus, *Ustilago maydis.* The Pep1 effector that was found in species across the Ustilaginaceae family including *Sporisorium reilianum*, *S. scitamineum*, *Melanopsichium pennsylvanicum,* and other *Ustilago* species (45.76–62.36% identity with the *U. maydis* Pep1) [106]. Pep1 is indispensable for pathogen virulence and is an inhibitor of apoplastic plant peroxidases, suppressing the oxidative burst. Cce1 is another core effector of *Ustilago maydis* of unknown functionality that is indispensable for infection; Cce1 is conserved among other smut fungi showing a range of sequence identity between 59–65% [107]. In *U. maydis*, a substantial number of its effectors are core effectors; 202 of the 467 effector candidates have an ortholog in the related smut pathogens [108].

*Colletotrichum* fungi represent another interesting model for effector conservation. CgEP1 was initially identified in the maize anthracnose pathogen, *Colletotrichum graminicola*. This protein is synthesized during the early stages of disease development and targets the host’s nucleus. EP1 arose from a gene duplication event in an ancestor of *Colletotrichum* sp. and has been under positive selection, resulting in CgEP1 homologs in several species of *Colletotrichum* sp. [109]. A recent inventory of effectors in *Colletotrichum* sp. genomes revealed 288–608 effectors per genome; cluster analysis of the effectoromes revealed that ~20% of conserved effectors were core effectors present in all *Colletotrichum* species [110]. Some examples of conserved effectors are NIS1, EC92, DN3, EC2-1, CEC2-2, CEC3, and CEC6, among others [110,111,112]. The core effector, NIS1, is not only found throughout the *Colletotrichum* genus but is broadly conserved in Ascomycota and Basidiomycota filamentous fungi [41]. In opposition to the usual effector narrative, most effector candidates of *Colletotrichum* species are conserved; only 4.1–15.6% of their effectoromes consist of species-specific effectors [110], giving support to the concept that each effectorome is a function of species host range and virulence.

It appears that not all shared effector homologs among species have the same functions, rather, some have evolved to have additional or different functions to suit their organism’s needs. Avr4 is a fungal effector and chitin-binding lectin that protects fungal cell walls against plant chitinases [55]. It was first described in *Cladosporium fulvum*, the causal agent of the leaf mold of tomato. Homologs of this protein were identified in *Pseudocercospora* (previously *Mycosphaerella*) *fijiensis*, and in *Cercospora beticola*, *C. nicotianae*, *C. apii*, and *C. Zeina* [113], *Dothistroma septosporum* [114], and *Pseudocercospora fuligena* [115], all of which are related Dothideomycete fungi. In addition to its role as an avirulence protein, the Avr4 of *C. flagellaris*, the causal agent of *Cercospora* leaf blight (CLB) on soybean, was found to be involved in cercosporin biosynthesis, since the Avr4 knockout mutant had a dramatically reduced production of this toxin [116]. In *P. fuligena* it was found that a second copy (paralog) of Avr4, called *Pf*Avr4-2, does not bind chitin but it binds to highly de-esterified pectin to relax the plant cell wall structure with the help of secreted endo-polygalacturonases to facilitate pathogen entry [117]. Another effector first described in *C. fulvum*, Ecp2, has homologs in closely related fungi, like *P. fijiensis* and *Mycospharella graminicola* [113], but the function of this effector is unknown. As more genome sequencing and effector identification studies are performed, we are likely to see more effectors being present across related and even unrelated species and genera. The previous effector families are shared among phylogenetically close relatives. The next examples have larger phylogenetic distribution.

Ecp6 is a secreted LysM-containing effector first described in *C. fulvum*. Ecp6 binds chitin oligosaccharides that are released upon degradation of the fungal cell wall, avoiding detection by the host chitin receptors [118]. Proteins with LysM domain are conserved throughout fungal taxa and evasion of immune detection by Ecp6s may very well be a common strategy of fungi to subvert host immunity [119]. RALPH and MAX constitute large effector families that were more recently discovered. The former, “RNAse-Like Proteins Associated with Haustoria” (RALPH), comprises 25% of the predicted effectors in *Blumeria graminis* [120], meanwhile MAX or “*Magnaporthe* Avrs and ToxB like” effectors were discovered by comparative 3D modeling of *Magnaporthe oryzae* effectors and effectors of the phylogenetically distant pathogen *Pyrenophora tritici-repentis* [121]. To date, they represent between 5 to 10% of the *Magnaporthe oryzae* and *M. grisea* effectoromes [122]. Functional analysis has validated that these proteins induce clear necrosis in *Nicotiana benthamiana* when co-expressed with the corresponding ‘cognate’ resistant protein.

ToxA, first identified in *Pyrenophora tritici-repentis,* has homologs in *Parastagonospora nodorum*, and *Bipolaris sorokiniania*, among other species, that most likely originated through horizontal transfer of these genes [123]. Similarly, AvrLm6 first reported in *Leptosphaeria maculans*, has been reported in *Leptosphaeria biglobosa*, *Fusarium oxysporum*, *Colletotrichum* sp., *Venturia inaequalis* and *V. pirina* [90]. Lastly, a Crinkler effector candidate CRN13, from the legume root pathogen *Aphanomyces euteiches*, is also found in the genome of the amphibian fungal pathogen, *Batrachochytrium dendrobatidis*. The effector was found to have detrimental effects on plants through the inhibition of root growth and produced abnormal growth in frog embryos [124]. The number of conserved effector families is still small, but the use of common domains during effector identification and 3-D homology modeling has undoubtedly improved our ability to predict effectoromes and will enable us to identify new effector families shared in even taxonomically distant microorganisms. Table 1 presents different levels of conservation found in effectors, from species-specific to widely distributed conserved effectors.

## 5. Effector Targets: Beyond the Apoplast

**Figure 2:**
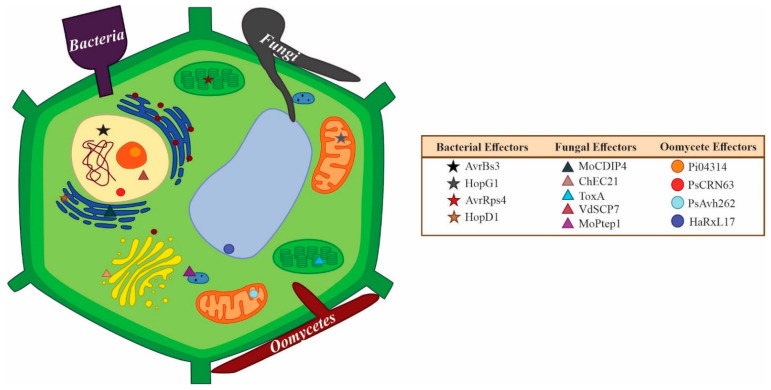
Examples of effectors and their intracellular targets. Secreted effectors enter the cell apoplast before they reach the interior of the cell; some are retained in the apoplast if their targets are apoplastic while others traverse the cell membrane to reach their targets inside the cell. In the intracellular bacterial effector examples given, the effectors AvrBs3 [159], HopG1 [160,161], AvrRps4 [86,162], and HopD1 [163] target the nucleus, mitochondria, chloroplast, and endoplasmic reticulum (ER), respectively. The fungal effectors displayed are AvrSr35 [164], ChEC21 [156], ToxA [165], VdSCP7 [166], and MoPtep1 [167] which target the ER, Golgi, chloroplast, nucleus, and peroxisomes, respectively. The oomycetes effectors Pi04313 [150] and PsCRN63 [168,169] target the nucleus while PsAvh262 [170] targets the mitochondria and HaRxL17 [171] associates with the plant tonoplast (vacuolar membrane).

The effector leaves the organism’s secretory system to arrive at either its cell wall, as in the case of those “protective effectors” (e.g., the ortholog of Avr4, PfAvr4 [145]), or is secreted into the extracellular space where it finds its host target in the apoplast or in the cytoplasm. Inside the cell, effectors may target various intracellular organelles (Figure 2). The haustorium is associated with fungal effector delivery [146], though effectors have been found to be secreted from conidia [147], as well as appressoria [130]. In pioneer investigations between 1996 and 2008 in fungal effector biology, the effectors that were identified and characterized were mainly apoplastic, like the Avr and Ecp effectors from the biotrophic fungus *Cladosporium fulvum* [5,30,48]. Regarding fungi and oomycetes, 314 protein effectors have been characterized to date, 228 from fungi, and 86 from oomycetes (compilation from the literature by Carreón-Anguiano et al., submitted). Recently, 176 known effectors were classified; 64 apoplastic (50 from fungi and 14 from oomycetes), and 112 cytoplasmic effectors (77 from fungi and 35 from oomycetes) [74].

The way in which effectors arrive at their targets is an interesting and controversial facet of effector biology. Canonical secretion in eukaryotes occurs through the endoplasmic reticulum and Golgi apparatus, then across the cell membrane via exocytosis of the secreted vesicles of the Golgi. Secreted proteins commonly have signal peptides: protein-sorting signals that direct the protein to the cell’s protein secretion machinery [148]. Conversely, proteins that lack signal peptides are usually considered to have non-canonical secretion and can be secreted by vesicular and non-vesicular pathways; vesicular pathways involve autophagy-based secretion and Golgi-bypass proteins while non-vesicular secretion includes protein translocation across plasma membranes and secretion by ABC-transporters [149]. It is not well known which of these pathways, if any, are used for non-canonical secretion of effectors, only that some effectors lacking predicted N-terminal signal peptides do exist and are secreted to the extracellular space. In the oomycete, *Phytophthora infestans*, a cytoplasmic effector lacking a signal peptide, Pi410314, was found to exhibit non-canonical secretion as Brefeldin A inhibition assays did not inhibit the retrograde secretion of the effector, compared to an apoplastic effector studied which showed canonical ER-Golgi secretion [150]. PsIsc1 of *P. sojae* and VdIsch1 of *Verticillium dahliae* are effectors that lack predicted N-terminal signal peptides. The effectors were mutated to remove the entire N-terminal region and as a result, both were found to have markedly reduced virulence in cotton seedlings indicating that putative unconventional secretion signals located in the N-terminal region of the proteins are necessary for their secretion [86]. Probably the most well-understood non-canonical secretion occurrence is in *Magnaporthe oryzae*, where cytoplasmic effectors were found to be secreted non-canonically by a form of secretion involving the exocyst complex and t-SNAREs [151] while apoplastic effectors such as BAS4, BAS113, and SLP1 are secreted by the conventional ER-Golgi pathway [152]. In the case of oomycetes, motifs in their sequences like the N-terminal RxLR-(d)EER motif mediate host cell entry by binding to phosphatidylinositol phosphates on the outer surface of plant plasma membranes [153]; the RXLR motif has since been identified in some fungi [154]. Other sequence features allowing effector movements are chloroplast/mitochondrial transit peptides and nuclear localization signals. These sequence features are native to plant proteins, and it is hypothesized that through mimicry, effectors would have evolved with these transit peptides to reach organelles such as the chloroplast [155].

Large-scale effector visualization has been achieved using fluorescent tags in *Colletotrichum higginsianum*; three effectors were targeted to plant peroxisomes, three others to plant cortical microtubules, and one to the Golgi apparatus, along with nine nuclear-targeted effectors [156]. In *Melamspora larici-populina*, effectors localized to the nucleus, chloroplast, and mitochondria [155]. In the oomycete, *Plasmopara viticola*, twenty-nine effectors were found to localize to the nucleus, nine to the cell membrane, three to chloroplasts, and one that targeted both chloroplast and mitochondria [157]. As more localization studies are undertaken, we will most likely see more effectors targeting all organelles in the plant host although the most common organelles targeted appear to be the nucleus and the cell membrane. Table 2 presents examples of effectors which have been proven to localize to different plant cell organelles. In a comparative study of effector targets of bacteria, oomycetes, and fungi, it was found that 95% of bacterial effector targets are in the cell membrane, nucleus, and/or cytoplasm while this value decreases to 63% for oomycetes that show more diversity in their targets (inclusive of peroxisomes and endoplasmic reticula). With respect to fungi, it was found that 61% of effector targets are cytoplasmic and are involved in signaling and protein processing, while signaling and transcription are more prevalent bacterial targets. The previously mentioned targets as well as those linked to metabolism are the main targets of oomycetes [158].

With respect to effector targets, some effectors have been found to have more than one target in an organism, like AvrLm4-7 of *Leptosphaeria maculans* which interacts with multiple R genes in *Brassica napus* [187]. On the other hand, a target may interact with multiple effectors of the same pathogen like the RGA4/RGA5 receptor pair in rice that is recognized by AVR-Pia and AVR1-CO39 of *Magnaporthe oryzae* [139]. Furthermore, one target may have interactions with several effectors of different organisms; the Rcr3 protease of tomato is targeted by the Avr2 effector of *Cladosporium fulvum* and two other effectors, EPIC1 and EPIC2B of *Phytophthora infestans* [188]. These targets in the host that attract many effectors have been denominated ‘hubs’ and are central in plant protein-protein interaction networks [189,190]. Common hubs include the serine/threonine protein kinase and mitogen-activated protein kinase families which are actively involved in plant immune signaling, transcription factors, enzymes involved in the biosynthesis or regulation of jasmonic acid and salicylic acid pathways, and other phytoregulators involved in host responses [191]. Similarly, common proteins or hubs in pathogens have been identified in interaction networks, for example, ubiquitin-like activating enzymes, small GTPases such as Rho, Ran, and Ras, SUMO-conjugating enzymes, thioredoxin reductase, among others [192]. Table 3 highlights examples of hubs in plant hosts and pathogens. Recently, through mining deduced proteomes and conducting interactomics analyses, multiple targets of effectors from different kingdoms (bacteria, fungi, oomycetes, and nematodes) were identified in *Arabidopsis thaliana* [193]. The existence of these common interactors demonstrates how pathogens evolved to cleverly debilitate their hosts, and in this manner, maintain low fitness costs as compared with the individual gene-for-gene interactions [189]. More high-throughput interactomics network studies are necessary to elucidate effector targets [194,195,196].

## 6. Effector Nature: The Rise of Non-Proteinaceous Effectors

The majority of effectors reported are small (<300 aa), secreted proteins (SSPs) which make up around 2–3% of the total proteome of fungal organisms [10]. Secondary metabolites and small RNAs (sRNA) have also been reported to suppress PTI and thus act as effectors. Here, the roles of these molecules are explored with the spotlight on plant-pathogen interactions.

### 6.1. RNA Effectors

RNA effectors discovered to date belong to the class of small silencing RNAs (sRNAs). These are short, non-coding RNAs capable of gene expression regulation through binding to host Argonaute (AGO) proteins and directing the RNA-induced silencing complex (RISC) to RNAs with complementary sequences [202]. In a pioneer study involving the fungal pathogen, *Botrytis cinerea,* sRNAs were found to bind to the argonaute protein 1 (AGO1) of the RNAi machinery in *Arabidopsis* to commandeer the host’s RNA-interference machinery and induce gene silencing of host targets. These sRNAs were able to silence transcripts of mitogen-activated protein (MAP) kinases such as mitogen-activated protein kinase 2 (MPK2) and MPK1 which are responsible for cell signaling to activate defense responses against pathogens. Transgenic *Arabidopsis* containing these pathogen sRNA effectors were more susceptible to the disease than the wild-type control plants, proving that these sRNAs play a role in the suppression of host immunity [34]. A more recent study showed that *B. cinerea* sRNA, Bc-siR37, targets the transcripts of multiple host proteins in *Arabidopsis* to subvert plant immunity [35]. The pathogen *Puccinia striiformis* f. sp. *tritici* (Pst) through the microRNA-like RNA 1 (Pst-milR1) attenuates wheat immunity by silencing the pathogenicity-related protein 2 (PR2), a protein that contributes to wheat resistance against the virulent Pst isolate [203]. This phenomenon of sRNA effectors translocating from the invader to the plant host to induce gene silencing of one or multiple targets is referred to as cross-kingdom RNA interference [204,205] and can be bidirectional when plants, in turn, release RNAs in their defense [206,207]. Pathogens, in response, have evolved effectors to suppress the RNA-mediated immunity of plants; *Arabidopsis* siRNAs that silence pathogen genes are inhibited by the *Phytopthora* protein effector PSR2 which interferes with host dsRNA processing [208].

RNA effectors have also been identified beyond the plant-pathogen interaction. The entomopathogenic fungus, *Beauveria bassiana*, uses a microRNA-like effector called bba-milR1 to silence the mosquito Toll receptor ligand Spätzle 4 (Spz4) upon host penetration to facilitate infection [202] and in the same interaction, it was later discovered that mosquitoes also use microRNAs (miRNAs) that translocate to fungal hyphae and target virulence genes in a fascinating example of insect-fungus cross-kingdom RNAi [209]. In silico RNA effector prediction is likely to gain popularity among researchers with the advent of bioinformatic pipelines for RNA effector identification [210], and high-throughput small RNA sequencing for the determination of sRNAs and their targets involved in host immunity [211,212,213,214].

### 6.2. Secondary Metabolite Effectors

Secondary metabolites (SM) are low molecular weight compounds that are not essential for growth but are still advantageous to the producer since they indirectly contribute to the survival and adaptation of the organism in its ecological niche [215,216]. Many fungal SMs are derivatives of polyketides, non-ribosomal peptides, hybrid polyketide–non-ribosomal peptides, and terpenes [217,218]. The genes responsible for SM production are usually found together in large genomic clusters and are coregulated [219]. Some of the core SM biosynthetic enzymes are polyketide synthases (PKSs), non-ribosomal peptide synthases (NRPSs), prenyltransferases (PTs)/terpene synthases (TSs), and dimethylallyl tryptophan synthases (DMATSs) [220,221].

Effectors that are secondary metabolites have a role in disease establishment and either induce necrotrophy [host selective toxins (HSTs) and non-HSTs] or do not induce necrosis but contribute to pathogen virulence [221]. Necrotrophs tend to have a larger proportion of SM biosynthesis genes in their genomes than biotrophs [222]. Necrotroph HSTs are involved in the inverse gene-for-gene interaction: a phenomenon occurring in a susceptible host where the HST effector is recognized by the host susceptibility gene product (S), triggering host cell death which is beneficial to the necrotroph. The most characterized SM effectors have been identified from *Cochliobolus* sp., host-specific necrotrophic fungi that target maize and use HSTs like T-toxin, victorin, and HC-toxin to establish disease on susceptible hosts [220]. T-toxin is a polyketide produced by *Cochliobolus heterostrophus* which targets the mitochondrial protein, URF13, resulting in the formation of pores in the mitochondrial membrane and necrosis [223,224]. A non-HST secreted by the hemibiotroph, *Pseudomonas syringae*, called coronatine (COR) (a hybrid between a polyketide, coronafacic acid (CFA), and a derivative of isoleucine, coronamic acid), suppresses salicylic acid (SA)-dependent defense responses and is a virulence factor for disease development in *A. thaliana*. In order to facilitate colonization, the toxin mimics one or more plant jasmonates involved in jasmonate signaling which antagonizes SA-dependent defense responses [225]. Some pathogen SMs have a positive contribution to plant health in the presence of a resistant host, for example, the Ace1 polyketide produced by the hemibiotroph, *Magnaporthe grisea*, which triggers resistance when recognized by the rice Pi33 resistance protein [226].

In silico genomics analyses of the SM gene clusters in pathogenic fungi have revealed the proportions of these core enzymes and their homologs in other fungi [219,227] and transcriptomic studies have revealed potential SM effectors that are upregulated in interaction with the host [218,228], but the characterization of the SMs has been a challenge due to difficulties in isolating metabolites found in small quantities, the need for suitable solvents, the limited availability of standards and limited mass spectra and NMR databases [229,230]. In addition, in silico reconstructed microbial SM pathways are sometimes incomplete, either because the genome in question has not been completely sequenced or because the microorganisms share part(s) of the pathways but have different end products [231,232,233] and to investigate and clarify these possibilities is not trivial work.

## 7. Effectors: Not Just Plant-Pathogenic Molecules

Until recently, the effector narrative has been centered on pathogenicity; effectors commonly being described as pathogen proteins and small molecules [3,59]. Pathogenic interactions between plants and microorganisms such as *Cladosporium fulvum*-tomato [234,235]; *Phytophthora infestans*-potato [236,237], the *Pseudomonas syringae* species complex with various hosts [238,239], are prime examples of well-studied pathosystems utilized for fungi, oomycetes, bacteria, respectively, and are also the leading pathosystems in effector biology studies. A budding area of interest centers on effectors beyond the plant-pathogen interaction, with discoveries in non-pathogenic organisms such as mutualist mycorrhizal species [13,186,240] and endophytes [16,241,242] in interaction with the plant host. These organisms also modulate host immunity with the help of effectors to establish plant-beneficial interactions.

One of the more noteworthy discoveries in effector biology has been the role of effectors in microbial interactions. In these interactions, effectors are required to subdue microbial competitors and dominate their respective niches [243]. Literature supporting the evidence of effectors in microbial interactions can be found for bacteria, especially effectors from the type VI secretion system that antagonize other bacterial and fungal competitors [244,245,246,247]. In fungal interactions, examples of effector candidates can be found in mycoparasitic interactions involving *Trichoderma* sp. against *Rhizoctonia solani* [248], *Pseudomyza flocculosa* against *Blumeria graminis* [147], and *Pythium oligandrum* against *Phytophthora infestans* [249].

**Figure 3:**
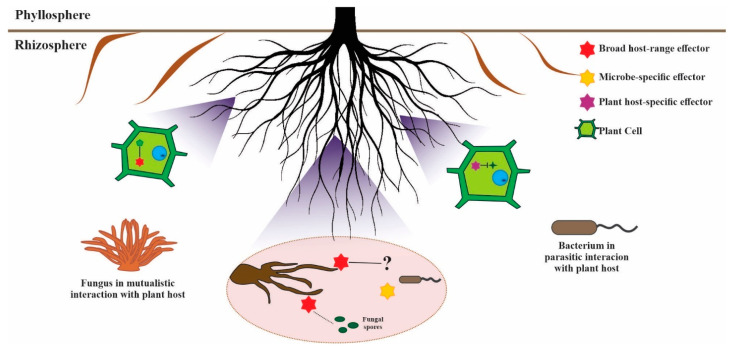
Effectors showing different host-range preferences based on the effector classification proposed by Snelders et al. (2018) [43]. The red effectors represent broad-host range effectors that are used by the mutualistic fungus against the plant host (far left) and against a bacterial competitor (center). It is unknown whether the red effector’s target in the bacterial competitor is the same target in the plant cell (large green icons) and the bacterium in response secretes a microbe-specific effector against the fungus (yellow effector). The purple effector represents a plant host-specific effector used by the bacterium (far right) that has an inhibitor function on the target (dark green star) in the plant cell.

Due to effector findings in interactions beyond the plant host, it has been postulated that effectors can be additionally classified based on their host specificity: effectors that target only plants, effectors that target only microbes, and effectors that target both plants and microorganisms [43] (Figure 3). Possible broad host-range effectors were identified in *Trichoderma atroviride* and *T. virens*, where expression of candidate effector genes (LysM protein, Epl2, and a hydrophobin, Tvhydii1) was observed in the presence of the plant *Arabidopsis thaliana*, as well as, during in vitro interaction with the fungus *Rhizoctonia solani*. In the same study, a metalloprotease was found to only be induced in the interaction with *R. solani* but not with the plant, a possible example of a host-specific effector [248]. Conversely, *T. atroviride’s* Tal6 is a broad host-range effector acting in microbe-microbe and microbe-plant interactions [250]. Tal6 is an effector with LysM domains that allow it to bind to chitin from the fungal cell wall, serving as protection against plant chitinases and interfering with the perception of its chitin fragments that could trigger a host immune response. The gene was upregulated during contact with the phytopathogen *R. solani* and Tal6 deletions mutants showed a decrease in antagonism against *R. solani*, *B. cinerea*, *Sclerotium cepivorum* and *C. lindemuthianum,* while overexpression of Tal6 results in increased antagonistic capacity [250]. Although the exact function of the Tal6 gene in microbial interactions was not elucidated, it may similarly act in hyphal protection against microbial chitinases as it does with plant chitinases.

Another addition to the short list of effectors discovered in microbe-microbe interactions is Zt6, a ribonuclease effector of *Zymoseptoria tritici*. This effector is detrimental to the plant host *Triticum aestivum*, the non-host *N. benthamiana*, as well as the bacterium *E. coli* and the yeasts *Saccharomyces cerevisiae* and *Pichia pastoris* during in vitro assay [201]. Recently, the functions of two effectors, VdAve1 and VdAMP3 of the fungal pathogen, *V. dahliae*, have been characterized in microbial interactions [251]. In plant-pathogen interaction, VdAve1 is an avirulence effector that is recognized by the receptor Ve1 in tomatoes. In microbial interaction, this effector was shown to selectively inhibit the growth of gram-positive bacteria in vitro. Analysis of the bacterial communities 10 days after in planta infection with *V. dahlia* wildtype and VdAve1 deletion mutant strains revealed significant differences in the microbiomes present; for example, bacteria of the Sphingomonadaceae family were completely absent in cotton plants infected with the wildtype fungus, meanwhile, they were present where the mutant was used. The direct application of the purified VdAve1 protein on the bacteria produced the same result, ruling out indirect effects of the effector on the host [251]. Conversely, VdAMP3, allows the fungus to suppress other fungi in decomposing plant tissue, while it forms its resting structures called microsclerotia, the ultimate phase of its disease cycle. The effector showed potent and specific antimicrobial activity against Saccharomycete and Sordariomycete filamentous fungi and yeasts, while bacteria are suppressed to a much lesser degree [252]. However, VdAMP3 does not appear to contribute to infection of the plant, *N. benthamiana*, since the *V. dahliae* mutant lacking the effector gene remained virulent, suggesting this effector is specifically employed in microbe-microbe interactions. The identification of microbial antagonism-related effectors opens a new avenue to produce novel antimicrobials as alternatives against recalcitrant pathogens which display resistance against the current antimicrobials. These effectors may prove useful not only for plants but additionally, for human health.

## 8. Discussion: Coming Changes to Effectoromics

As highlighted in this review, the initial beliefs about effectors have constantly been evolving with novel effector discoveries. This is the first time such a range of effector characteristics has been comprehensibly presented in the context of past and present knowledge related to effectors. It is also uncommon to witness within the effector literature, varied types of effector classifications apart from the common classification of localization in the plant host (apoplast or cytoplasm).

The dogmas in effectoromics seem as though they are meant to be challenged (Table 4) and as such, the effector literature merits a change in how effectors are defined. Effectors are often referred to as *virulence-associated molecules* due to their role in disease development; they either induce susceptibility or resistance depending on the presence of related targets in the host and as such, can have a dual function as promoters of disease or health. The proverbial box in which effectors were first placed is being dismantled as these molecules have been found beyond the limits of pathogenicity and virulence, although the socioeconomic impact of disease merits the attention paid to effectors in plant-pathogen interactions. Ultimately, effectors should be referred to as *interaction-associated molecules* since they are used by both pathogenic and non-pathogenic organisms.

The study of effectors in microbe-microbe interactions and in tripartite plant-pathogen-mutualist interactions have revealed effectors involved in mycoparasitism and biological control of pathogens in the host [201,248,253], as well as the induction of disease resistance in plants [254,255]. Microbial effector-based screening may facilitate the selection of elite strains of antagonists that can be applied to the field. It also forms the basis for the development of novel bioproducts for exogenous application to plants. Effective effector-bioproducts may be used to trigger systemic resistance or help modulate the composition of plant microbiomes for plant health promotion. One of the biggest limitations in understanding effector molecules in microbe-microbe interactions is the technical barrier associated with elucidating their targets and their localization in vivo.

Investigations into the host-pathogen interactomes are identifying core proteins that are suitable targets for engineering disease resistance [104,105], and novel algorithms/pipelines for identification of proteinaceous and non-proteinaceous effectors are elucidating complete pathogen effectoromes providing a sound foundation for effectoromics-based pathogen control strategies [69,72,73,81,210]. To date, the number of effector families is still small, but the construction of novel algorithms that allow us to expand and compare effectoromes may result in the identification of conserved effector families. The identification and characterization of conserved effectors among pathogens (core effectors) may provide effective solutions to disease management through their inactivation in the field.

Another necessary revolution in effectoromics involves the elucidation of the true effectorome; the identification of non-canonical effectors will be essential for the expansion of in silico-identified effector sets where the implementation of common effector domains and motifs are particularly useful for this type of effector. More occurrences are likely to be revealed of domains and motifs shared between fungi and oomycetes that were previously described as unique to each kingdom e.g., the RXLR and CRN motifs that are associated with oomycetes and LysM, ceratoplatanin, ribonuclease/ribotoxin, etc. domains commonly associated with fungal effectors. Additionally, the identification of novel motifs among the different kingdoms represents a promising tool that will promote effectorome elucidation.

## 9. Conclusions

Locating the resistance proteins targeted by Avr effectors in plant hosts has been a priority in effectoromics research for effector-assisted plant breeding [256,257,258], but varied possibilities for disease management exist with effector molecules [259]. This review highlighted many changes which have occurred in the study of fungal effectors since the gene-for-gene model. Effector research, especially in the last 30 years, has revealed the dynamic nature of effectors in different interactions, with and beyond the plant host. There are effectors which exist of varying molecular natures, functions, localizations in the host cell, and interactions (with one or more hosts and targets). Investigations surrounding effector targets beyond the R gene and how they can be utilized in plant protection should dominate effectoromics in the coming years; whether a particular effector has the same target in different hosts is an important line of investigation to be taken into consideration. In addition to the changes to the field mentioned in this review, others are on the horizon and are sure to improve our current understanding of what effectors are and how they can be applied to our benefit. Successful times are in sight for effectors in biotechnology.

## Figures and Tables

**Table 1:** Effectors can be species-specific, genus-specific or conserved across various genera.

Distribution *	Effector Type	Organism	Effector Name and Uniprot ID	Function	Reference
Conserved: Different classes in Ascomycetes	Ceratoplatanin family	*Botrytis* *cinerea*	BcSpl1 (A0A384JBC5)	Phytotoxin, elicits systemic resistance (SAR) in tobacco. Necrosis (HR) in tobacco, tomato and *Arabidopsis*	[125,126]
Conserved: Wide distribution in Dothideomycetes class (Ascomycetes)	LysM domain	*Cladosporium* *fulvum*	Ecp6 (B3VBK9)	Virulence factor; binds to fungal chitin to prevent chitin triggered-immunity in host	[118]
Conserved: *Colletotrichum* genus (Glomerellales order), *Fusarium, Trichoderma,* (Hypocreales order) in Sordariomycetes class. Few species in Dothideomycetes class (Ascomycetes)	Necrosis inducing protein	*Colletotrichum* *orbiculare*	NIS1 (H7CE97)	Suppresses PAMP-triggered immunity, targets kinases BAK1 and BIK1 Recognition is followed by a plant cell death response (necrosis), potential avirulence effects	[41]
Conserved: Few genera in Sclerotiniaceae family (Ascomycetes phylum)	Necrosis and ethylene-inducing proteins (NLPs)	*Botrytis* *cinerea*	BcNep2 (Q079H3)	Induces cell death in dicotyledonous plants	[127]
Conserved: *Fusarium* genus (Hypocreales order), *Bipolaris* genus (Pleosporales order), *Verticillium* genus (Glomerellales order), in Sordariomycetes class (Ascomycetes phylum)	Necrosis and ethylene-inducing proteins (NLPs)	*Fusarium* *oxysporum* f. sp. *erythroxyli*	NEP1 (O42737)	Induces necrosis and ethylene emission in *Erythroxylum coca* leaves	[128,129]
Conserved: Many species in *Colletotrichum* genus (Glomerellales order) and *Fusarium* genus (Hypocreales order)	Necrosis inducing protein	*Colletotrichum higginsianum*	ChNLP1 (K7N7F9)	Induces necrosis in *N. benthamiana*	[130]
Conserved: Many species in Mycosphaerellaceae family, Dothideomycetes class	Chitin-binding type-2	*Cladosporium* *fulvum*	Avr4 (Q00363)	Triggers a Cf-4-mediated hypersensitive response (HR) in tomato; protects fungal cell walls against hydrolysis by plant chitinases	[55,113,131]
Conserved: Species in Ustilaginaceae family	Pep1	*Ustilago* *maydis*	Pep1 (G0X7E8)	Inhibitor of plant peroxidases; targets maize peroxidase POX12 to suppress plant immunity	[106,132]
Conserved: Few species in Magnaporthales, Sordariales, Xylariales, Hypocreales, Glomerellales orders	MC69	*Magnaporthe* *oryzae*	MC69 (L7JHY1)	Development of invasive hyphae affected in mc69 mutant; reduced pathogenicity on host	[133]
Specific to genus	Avr	*Phytophthora* *sojae*	PsAvh163 (G1FRR2)	Suppresses PTI and ETI responses in Arabidopsis Activates immunity (HR response) in the *Nicotiana* genus.	[134]
Specific to genus	Hop	*Pseudomonas* *syringae*	HopAl1 (Q888W0)	Phosphothreonine lyase; inactivates MAPKs in *Arabidopsis* to overcome PTI	[135]
Specific to genus	Suppressor of necrosis 1 SNE1	*Phytophthora* *infestans*	SNE1 (A2CLL0)	Suppresses cell death induced by NLP and Avr effectors in *N. benthamiana* and *Solanum* *lycopersicum*	[136]
Specific to genus	Avr	*Melamspora* *lini*	AvrM (Q2MV46)	Induces ETI in host; targets resistance protein M	[137,138]
Specific to genus	Avr	*Magnaporthe* *oryzae*	AvrPia (B9WZW9)	Induces ETI in host; targets resistance protein RGA5	[139]
Species-specific	Avr	*Cladosporium* *fulvum*	Avr9 (P22287)	Induces ETI in host; Targets resistance protein of Cf-9	[140]
Species-specific	Avr	*Magnaporthe* *oryzae*	AVR-Pik (C4B8B8)	Induces ETI in host; targets resistance protein Pik	[56,141]
Species-specific	Biotrophy-associated secreted (BAS) proteins	*Magnaporthe* *oryzae*	BAS1 (G5EHI7)	Triggers defense response in host; overexpression increases virulence, sporulation and reduces expression of host defense-related genes	[142,143]
Species-specific	Ribonuclease family	*Blumeria* *graminis*	BEC1054 (N1JJ94)	Ribonuclease like; binds to host ribosomes and inhibits the action of plant ribosome-inactivating proteins (RIPs)	[144]

* Distribution was determined by Blastp at NCBI for fungi (taxid: 4751) and oomycetes (taxid: 4762).

**Table 2:** Examples of intracellular effectors with experimentally proven locations.

Effector	Cell Localization	Microorganism	Kingdom	Lifestyle	Reference
CgEP1	Nucleus	*Colletotrichum graminicola*	Fungi	Hemibiotrophic	[109]
ChECs (nine)	Nucleus, peroxisomes, and microtubules	*Colletotrichum higginsianum*	Fungi	Hemibiotrophic	[156]
MoCDIP4	Mitochondria	*Magnaporthe oryzae*	Fungi	Hemibiotrophic	[172]
Rab8a	Golgi apparatus	*Phytophthora infestans*	Oomycete	Hemibiotrophic	[173]
CSEP0064/ BEC1054	Cytoplasmic	*Blumeria graminis*	Fungi	Biotrophic	[144]
Sntf2	Chloroplast	*Colletotrichum gloeosporioides*	Fungi	Hemibiotrophic	[111]
CgNLP1	Nucleus	*Colletotrichum gloeosporioides*	Fungi	Hemibiotrophic	[174]
ToxA	Cytoplasm	*Parastagonospora nodorum*	Fungi	Necrotrophic	[175]
SsITL	Chloroplast	*Sclerotinia sclerotiorum*	Fungi	Necrotrophic	[176]
PEF1	Peroxisomes	*Magnaporthe oryzae*	Fungi	Hemibiotrophic	[177]
DspA/E	Peroxisomes	*Erwinia amylovora*	Bacteria	Necrotrophic	[178]
RsCRP1	Mitochondria and chloroplasts	*Rhizoctonia solani*	Fungi	Necrotrophic	[179]
RipAA	Chloroplasts	*Ralstonia solanacearum*	Bacteria	Necrotrophic	[180]
HopAF1	Plasma membrane and cytoplasm	*Pseudomonas syringae*	Bacteria	Hemibiotrophic	[181]
PcRxLR48	Nucleus	*Phytophthora infestans*	Oomycete	Hemibiotrophic	[182]
PsAvh52	Cytoplasm and nucleus	*Phytophthora infestans*	Oomycete	Hemibiotrophic	[183]
AvrLm1	Plasma membrane	*Leptosphaeria maculans*	Fungi	Hemibiotrophic	[184]
PstGSRE4	Nucleus	*Puccinia striiformis* f. sp. *tritici*	Fungi	Biotrophic	[185]
MiSSP7	Nucleus	*Laccaria bicolor*	Fungi	Ectomycorrhizal symbiont	[186]
RsCRP1	Mitochondria and chloroplasts	*Rhizoctonia solani*	Fungi	Necrotrophic	[179]
PexRD54	Golgi apparatus	*Phytophthora infestans*	Oomycete	Hemibiotrophic	[173]

**Table 3:** Examples of hubs (core proteins commonly involved in plant-pathogen interactions) identified in hosts and pathogens.

Plants				
Host Target (Hub) (Targets in Different Hosts Targeted by Multiple Effectors)	Function in the Host	Targeted by (Selected Examples)	Microorganisms	References
Chorismate mutase (CM)	Salicylic acid (SA) biosynthesis	Cmu1, Mi-CM-3, AvrPtoB	*Ustilago maydis* (fungus), *Meloidogyne javanica* (nematode), *Pseudomonas syringae* (bacterium)	[197,198]
Isochorismatase (ICM)	Salicylic acid (SA) biosynthesis	VdIsc1, PsIsc1, HopI1	*Verticillium dahliae* (fungus), *Phytophthora sojae* (oomycete), *Pseudomonas* *syringae* (bacterium)	[197]
JAZ6, negative regulator of JA induced transcription	Jasmonic acid (JA) biosynthesis	MiSSP7, HopZ1a, HopX1	*Laccaria bicolor* and *Golovinomyces orontii* (fungi), *Hyaloperonospora* *arabidopsidis* (oomycete), *Pseudomonas syringae* (bacterium)	[197]
AuTophaGy (ATG) proteins	Autophagy	HrpZ1, HopF3, AvrPtoB, HrpZ1, HopO1-2,	*Bremia lactucae*, *Hyaloperonospora* *arabidopsidis* (oomycetes), *Uromyces fabae* (fungus), *Globodera pallida* (nematode), *Pseudomonas syringae* (bacterium)	[199]
**Pathogen**				
**Pathogen hub** **(Multiple targets to one effector)**	**Functions**	**Targets in the host**	**Microorganisms where that effector has been identified**	**References**
AvrPtoB	Interferes with salicylic acid (SA) biosynthesis and autophagy in plants	CM, ATG1	*Pseudomonas syringae*	[198,199]
Zt6	Phytotoxic and antimicrobial ribonuclease effector	Plant and microbial rRNA species	*Zymoseptoria tritici*	[200,201]
VdAMP2	Soil colonization (antibacterial activity), and induction of necrosis in *Nicotiana* *benthamiana*	Unknown	*Verticillium dahliae*	[17]
VdAMP3	Soil colonization (antifungal activity)	Unknown, mycobiome manipulation	*Verticillium dahliae*	[17]
VirD5	Stabilizes VirF for *Agrobacterium* infection	*Arabidopsis thaliana* AT1G09270.1 AT1G43700.1 AT3G06720.1 AT5G59710.1 VIP2	*Agrobacterium tumefaciens*	[193]
Avr2	Inhibits tomato Rcr3 protease	*Arabidopsis thaliana* AT1G47128.1 AT3G19400.1 AT3G45310.1 AT4G35350.1 AT5G60360.1	*Cladosporium fulvum*	[193]
HARXL106	Suppresses transcriptional activation of salicylic acid (SA)-induced defense genes	*Arabidopsis thaliana* AT1G32230.1 AT2G35510.1	*Hyaloperonospora* *arabidopsidis*	[193]
RXLR24	Inhibitor of RABA GTPase-mediated vesicular secretion of antimicrobial PR-1 and PDF1.2	*Arabidopsis thaliana* AT1G06400.1 AT1G09630.1 AT1G16920.1 AT2G30950.1 AT3G18820.1 AT3G46830.1 AT3G56940.1 AT4G18800.1 AT4G39990.1 AT5G45750.1 AT5G47960.1 AT5G59150.1 AT5G60860.1 AT5G65270.1	*Phytophthora brassicae*	[193]

**Table 4:** A summary of changes in concepts in the field of effectoromics.

PAST	PRESENT
Effectors show no or limited sequence homology or conservation but may share structural properties	Some effectors are homologs of effectors in other microorganisms. Some orthologs display a high level of sequence conservation
Homologs are distributed in close phylogenetic relatives	Some effectors are distributed in phylogenetically related and distant organisms (core effectors)
Effectors are small proteins (<300 or 400 amino acids)	Limit in length of known current effectors is ~ 850 amino acids
Effectors are secreted proteins with signal Peptides	Many lack signal peptides and are secreted by little-understood non-conventional processes
Effector proteins lack TMDs	Some true effectors have one or two TMDs; the current limit for TMDs in a true effector is 6
The majority of effectors initially discovered were extracellular, apoplastic proteins	Many effectors also target cytoplasmic and organellar host proteins. Some effectors may even target both apoplast and cytoplasm
Effector interactions follow the gene-for-gene model	Effector interactions encompass the gene-for-gene model, guard model, the decoy model, integrated decoy model etc.
Effectors are proteins (Enzymatic or non-enzymatic)	Effectors can be proteins, RNA or secondary metabolites
Effectors induce the hypersensitive response (local, visible lesion) on the host leaves	Not all effectors induce HR. Biotrophic effectors can interfere with downstream reactions triggered by effector-R cognate recognition and prevent development of visible lesions
MTI and ETI are independent immunity Pathways	MTI and ETI are interconnected
Effectors are produced by pathogens to target plant hosts	Produced by pathogens and non-pathogens with targets in a wide spectrum of organisms
Effector roles are associated with conquering the plant host	Effector roles documented in host and niche conquest; roles in shaping microbiomes and roles in reproduction and development of producer organism
Each effector has one target or cognate	Some effectors are multifunctional or have many different targets in the same host or different hosts; and vice versa, some host targets are targeted by multiple effectors from the same or different microorganisms
